# Logistical Challenges Associated With Caregiver Coordination of In-Person Medical Appointments

**DOI:** 10.1093/geroni/igaf122.908

**Published:** 2025-12-31

**Authors:** Lauren Moo, Victoria Ngo, Elizabeth Marfeo

## Abstract

Arranging to bring a medically complex older person to an in-person medical visit involves lots of planning and disruption of daily routines. Family caregivers coordinating and navigating such visit-associated logistics must incorporate such challenges into their already strained schedules, potentially adding to caregiver burden and burnout. Virtual visits may reduce some of these challenges, but these benefits are difficult to catalog as a formal assessment of these visit-associated logistics has not been previously undertaken. This symposium will include five talks. The first will describe qualitative data from interviews with caregivers of people living with dementia (PLWD) about their experience bringing the PLWD to medical visits, including preparing for visits and visit-related time and travel. The second will describe a novel visit-associated logistics (VAL) framework that includes 6 domains: scheduling, preparing for the visit, visit-related time and travel, caregiver independence, routine disruption, and overall feelings & impressions. The third talk will share VAL results from a web-based national survey of > 500 informal caregivers of older Veterans regarding the most recent in-person medical visit they helped their care recipient attend. The fourth talk will discuss the additional logistical challenges faced by caregivers coordinating medical visits for older adults living in rural areas. The fifth talk will describe the VAL assessment results from 20 family caregivers of PWLD in the context of their qualitative reflections on the merits of in-person versus video visits.

